# The Efficacy of Lidocaine Spray in Pain Relief during Outpatient-Based Endometrial Sampling: A Randomized Placebo-Controlled Trial

**DOI:** 10.1155/2018/1238627

**Published:** 2018-10-21

**Authors:** Wiphawee Luangtangvarodom, Densak Pongrojpaw, Athita Chanthasenanont, Junya Pattaraarchachai, Kornkarn Bhamarapravatana, Komsun Suwannarurk

**Affiliations:** ^1^Department of Obstetrics and Gynecology, Thammasat University, Pathum Thani, Thailand; ^2^Chulabhorn International College of Medicine, Thammasat University, Pathum Thani, Thailand; ^3^Department of Preclinical Science, Faculty of Medicine, Thammasat University, Thailand

## Abstract

Abnormal vaginal bleeding is one of the most frequent problems found in gynecology. Endometrial histopathology is needed for definite diagnosis. It was obtained either from endometrial tissue sampling or from standard uterine curettage. Office endometrial tissue sampling is an easy and low morbid procedure. It is usually associated with pain and discomfort. Topical anesthetic agent is needed for pain relieving. This study was conducted in outpatient gynecology clinic, Thammasat University Hospital, Thailand. It was a double blind randomized controlled trial. A total of 140 participants were enrolled in study and control group. Each group consisted of 70 cases. Study group received topical spray of 10% lidocaine (40 mg) before endometrial aspiration. Topical spray of 0.9% normal saline was performed in control group. Novak curettage was an application for endometrial tissue obtaining in this study. Visual analog scale (10cm-VAS) was used for pain evaluation. Demographic character of both groups showed no statistical difference. The percentage of participants who had severe pain (VAS≥7) during tenaculum application and Novak curettage insertion and during procedure were 28.5% (20/70) versus 12.9% (9/70), 55.7% (39/70) versus 38.5% (27/70), and 78.5% (55/70) versus 60% (42/70) in control and study group, respectively. Both groups had no significant differences of postoperative pain at 15 minutes and 2 hours. This study indicates that topical lidocaine spray can relieve pain during endometrial tissue sampling.

## 1. Introduction

Abnormal vaginal bleeding is a common presenting complaint of women aged 35 years or more at the gynecology clinic. It is a major cause of distress for patients as well as a sign of a serious gynecological condition. The causes of abnormal vaginal bleeding most commonly include endometritis, endometrial hyperplasia, myoma uteri, adenomyosis, and endometrial cancer. Therefore, it is important to find the cause of abnormal vaginal bleeding by pathological examination of the endometrial tissue.

The endometrial tissue can be obtained from either sharp or negative pressure aspirator. The instrument available includes Endocell®, Pipelle®, Karman®, or Novak. The Novak curettage is considered to be available tool for endometrial biopsy as it is quick and easy to use. The Novak endometrial curette is reusable and obtains an adequate amount of tissue for pathological examinations, even while the patient has bleeding. No need for sedation or analgesia was used before the procedure. It is made of stainless steel and comes in various sizes ranging from 1 to 4 mm in diameter [1]. Therefore, the gynecologist could choose the appropriate size of the Novak curettage to suit each individual patient. Another advantage of the Novak curette is that, during the procedure, the patient does not require general anesthesia or deep sedation, and thus the procedure is available in the outpatient department and no admission is required. Even in patients who experience heavy vaginal bleeding, the Novak curette is able to obtain adequate tissue for the pathological examination, due to its larger diameter compared to other instrument.

Lidocaine, an amide group local anesthetic drug [2], acts by inhibiting the influx of sodium into neural cell which results in the inhibition of the production and transmission of neurotransmitters [3, 4]. Lidocaine is available in many different forms such as sprays, injections and gel, and many concentrations including 1 %, 2 %, and 10 %. The lidocaine spray is a good option for local anesthetics as it is easy to use, quickly absorption within 1 to 5 minutes, and the analgesic effects last for 10 to 15 minutes [5].

Endometrial biopsy can cause patients moderate-to-severe pain during the procedure [6]. The study of pain during curettage is widely done with oral or neuroleptic anesthesia prior procedure, as well as local analgesic drugs, including both intrauterine infiltration and cervical injection [7–10]. On the other hand, some studies focused on pain during cervical or intrauterine procedures such as intrauterine device (IUD) insertion, hysteroscope, and loop electrosurgical excision procedure (LEEP) [11–15].

The objective of our study was to investigate the analgesic effect of lidocaine spray on the patient's perception of pain during endometrial biopsy. The focus group is women aged 35 years or more with abnormal vaginal bleeding.

Pain score was categorized in two groups, mild-moderate and severe. Mild-moderate pain is defined as a pain score of 0 to 6. Severe is defined as a pain score of 7 to 10. The results of both groups were calculated by chi-square test.

## 2. Methods

This randomized controlled trial was conducted at the outpatient clinic, Department of Obstetrics and Gynecology, Thammasat University Hospital, Pathum Thani, Thailand, between February 2017 and January 2018. The approval from the Ethics Committee on Clinical Research of Faculty of Medicine, Thammasat University, was obtained prior to the study (MTU-EC-OB-2-145/59) (TCTR20170117001). The inclusion criteria included patients 35 years or over who presented with abnormal vaginal bleeding at the gynecology outpatients department between the specified period. Patients who had a history of abnormal coagulopathy were currently taking medication affecting coagulation, were allergic to lidocaine, were pregnant, had current pelvic infection, had cervical stenosis, and had known cases of abnormal uterine structure were excluded from the study.

After personal counseling and filling the inform consent, a total of 140 participants were randomly assigned into a study and control groups using a computer-generated random number chart with a random block of four. Random allocation was done before curettage. The study group consisted of 70 participants. Patients in this group received 4 puffs (40 mg) of 10% lidocaine spray, prior to endometrial biopsy with the Novak curette. The control group (n=70) received 4 puffs of 0.9% normal saline.

Two spray bottles of similar external appearances were prepared and numbered; one of the bottles contained normal saline, while the other contained 10% lidocaine. An envelope containing the medication number on its cover was opened by the responsible nurse to reveal the randomization and the trial medication was given accordingly. The patients, nurses, and gynecologists who were involved in the procedure were blinded.

Four puffs of the spray containing either normal saline or 10% lidocaine were applied on the cervical surface 3 minutes before starting the procedure. After tenaculum application, the Novak curette was inserted through cervical os, and negative pressure was created via a 10-ml-syringe. Endometrial tissue was collected by aspiration and rotation at 3, 6, 9, and 12 o'clock. The patients were then asked to rank their pain on a 10 cm VAS score, from 0 (no pain) to 10 (worst pain). Six different times of pain evaluation were before the procedure, after speculum insertion, after tenaculum application and insertion of Novak curettage, during the procedure, at 15 minutes, and 2 hours after the procedure. After the procedure, if adverse outcome was immediately reported, patients would be kept in observation for 60 minutes.

*Statistical Analysis*. ThePearson chi-square test and Fisher exact test were used to compare pain score in both groups. The Student* t*-test was used to analyze the demographic data. The statistical package for the social science (SPSS Inc., Chicago, IL USA) for Windows version 17 was used for data analysis. Statistical significance refers to* p*–value < 0.05.

## 3. Result

One hundred and forty participants were assessed for eligibility. The age of the participants ranged from 37 to 67 years. Demographic characteristics of patients in both groups were comparable in age, body weight, height, BMI, parity, number of vaginal deliveries, number of abortions, and age of their last child. There were no statistically significant differences of any of the demographic data between both groups (Table 1). Predisposed illness of participants was reported in Table 1. Sixty-one percent of placebo group had no underlying illness while only 47 percent of treated group fell in this category.

All procedures were successfully completed without any severe complications or serious adverse reaction, hypotension, or syncope. Out of the 140 patients, 12 and 9 participants experienced mild complications in control and study groups, respectively, with no statistical significance. Mild complications included abdominal pain and vasovagal reaction such as nausea or dizziness. No further treatment was needed (Table 2). Average time required for the procedure was 6 minutes in both the control and study groups.

**Figure 1:**
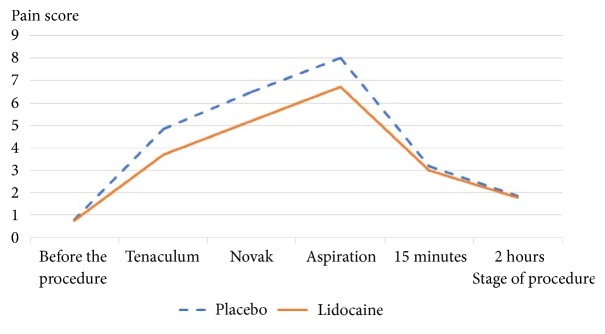
Mean pain score during various stages of procedure.

Pain scores collected during the procedure are presented in Figure 1. Pain score was significantly lower in lidocaine group than control group, when the tenaculum was applied, at the insertion of the Novak curette and during tissue aspiration at* p*<0.05. The ratio percentage of participants reporting severe pain between placebo and lidocaine spray treated groups during tenaculum placement, Novak curettage insertion, and tissue aspiration were 28.5 versus 12.8, 55.7 versus 38.5, and 78.5 versus 60, respectively, as seen in Table 3.

On the other hand, there were no statistically significant differences in the number of patients in the severe pain group between the placebo and the intervention arm at 15 minutes and 2 hours postoperatively. The number of participants in both study and control group in severe group at the end of procedure reduced from 55 and 42 to 8 and 7 at 15 minutes and to 3 and 1 at 2 hours, respectively.

## 4. Discussion

Abnormal uterine bleeding is an important issue in women aged 35 years or more. It is crucial to provide abnormal uterine bleeding patients with a definitive diagnosis, which can be obtained by endometrial biopsy. The endometrial biopsy can be performed in either an outpatient or an inpatient setting.

Outpatient-based endometrial biopsy can be performed by using many devices. The advantages of using these devices are that they do not require the use of general anesthesia. As a result, it is important to minimize the patient's pain and discomfort during the procedure by using other forms of analgesia. Cervical pain transmitted via the pelvic splanchnic nerve to central nervous system. Many local anesthetics and oral analgesic drugs were experimented to help reducing pain during endometrial biopsy [7, 8].

In the present study, lidocaine spray application at cervix significantly reduced pain during tenaculum application. The use of lidocaine spray has been previously studied during multiple gynecological procedures, with positive results. Karasu et al. showed that the use of lidocaine spray during IUD insertion reduced pain during both tenaculum application compared to placebo and paracervical block. Paracervical injection of lidocaine during IUD insertion only blocked the pain during IUD insertion period [12]. Karasahin's study showed that 10 and 20 mg of lidocaine spray could reduce pain score during cervical traction and contrast media injection in hysteroscopic procedure [16]. Karasahin et al. also reported the positive effect of lidocaine spray. Lidocaine injection plus lidocaine spray reduced mean pain score during surgical abortion significantly more than lidocaine injection only [17]. In consistent with our study, their pain score during tissue aspiration decreased significantly.

Aksoy et al. [8] reported the median pain score of placebo and study group whom used lidocaine spray during outpatient-based endometrial sampling. Aksoy's study was administered 40 mg of lidocaine spray, 30 mg to cervical surface, and 10 mg towards cervical canal. The results showed that the use of lidocaine significantly reduced the overall pain during the procedure and after operation compared to placebo (*p*<0.05).

Our study was performed pain evaluation in different stage of procedure. The result showed a significant reduction in pain score during tenaculum application and insertion of Novak curette and during tissue aspiration. Contrast to Aksoy's study, our postoperative pain was no statistically significant between both groups. We think that the similarity of pain level between both groups might be due to the short efficacy of lidocaine spray last for 10-15 minutes.

The 10 cm VAS system was used in this study to obtain a pain score at various points during the procedure. The pain score was then grouped into either mild-moderate (0 to 6) or severe (7 to 10) pain. This study showed that the number of patients in the severe pain group was significantly lower in the intervention arm using lidocaine spray compared to placebo in three measurements, during the application of the tenaculum and the insertion of the Novak curette and during tissue aspiration. Therefore, the application of lidocaine spray significantly lowered the overall pain the patient experienced during the procedure compared to placebo group (p<0.05). The operators recorded higher satisfaction performing the procedure in patients of the treated group. This might be from the fact that patients with lidocaine spray experienced less pain during the entire procedure.

The main limitation in this study was a subjective concept of pain among different patients [8]. Pain perception was varied from individual and personal experience. Nevertheless, this study showed positive result of lidocaine local anesthetic effect.

## 5. Conclusions

Our study sought to reduce pain during outpatient-based endometrial biopsy which might lead to better patient experience, reduce anxiety, and produce an overall better outcome. Our study showed that the application of topical lidocaine spray significantly reduced pain during the procedure when compared to placebo. Lidocaine spray has an advantage when compared with paracervical block, no risk of lidocaine intravasation, infection, or bleeding. It is easily to administer, worthily with cost-effectiveness, and comfortable. So, lidocaine spray is a suitable analgesic option during endometrial biopsy.

## Figures and Tables

**Table 1:** Demographic data of participants.

	placebo group	Lidocaine group	*p*-value
	*(N = 70)*	*(N = 70)*	
Age (year)*∗*	48.28 ± 7.48	47.41 ± 7.59	0.495
Body weight (kg)*∗*	62.84 ± 15.65	65.34 ± 19.52	0.405
Height (cm)*∗*	157.38 ± 5.92	157.28 ± 6.99	0.1
BMI (kg/m^2^)*∗*	25.31 ± 5.84	26.26 ± 6.9	0.384
Parity*∗*	1.72 ± 1.17	1.85 ± 1.31	0.543
Normal labor*∗*	1.08 ± 1.01	0.98 ± 1.14	0.586
Abortion*∗*	0.44 ± 0.6	0.38 ± 0.72	0.614
Age of last child(year)*∗*	12.88 ± 11.38	15.12 ± 10.71	0.232
Duration (minute)*∗*	6.94 ± 3.34	6.47 ± 2.4	0.34
No UD*∗∗*	43(61.4)	33(47.1)	0.09
DM*∗∗*	4(5.7)	6(8.6)	0.745
Heart*∗∗*	2(2.59)	0	0.496
HT*∗∗*	11(15.7)	15(21.4)	0.385
Anemia*∗∗*	1(1.4)	2(2.9)	1
Hormonal used*∗∗*	7(10)	15(21.4)	0.063
Hx of STD*∗∗*	4(5.7)	5(7.1)	1
Hx of cervical dilatation*∗∗*	21(30)	24(34.3)	0.587
Menopause*∗∗*	19(27.1)	20(28.6)	0.85

*∗*Mean ± SD (standard deviation), *∗∗* n (%), BMI: body mass index, UD: underlying disease, DM: diabetes mellitus, HT: hypertension, Hx: history, and STD: sexually transmitted diseases.

**Table 2:** Symptom reported by participants during and after procedures.

	placebo group	Lidocaine group	
	*(N = 70) (*%)	*(N = 70) (*%)	*p*-value
Abdominal pain	8(11.4)	4(5.7)	0.366
N/V	0	2(2.9)	0.496
Dizziness	4(5.7)	4(5.7)	1
Hypotension	0	0	1
Syncope	0	0	1

N/V: nausea and vomiting.

**Table 3:** Comparison of pain scores in the studies population.

	Placebo group		Lidocaine group		
	*(N = 70) (*%)		*(N = 70) (*%)		
VAS	severe	mild	severe	mild	*p*-value
Before the procedure^a^	2(2.8)	68(97.1)	1(1.4)	69(98.5)	0.559
After Tenaculum was placed^b^	20(28.5)	50(71.4)	9(12.8)	61(87.1)	0.022*∗*
Insert Novak curettage^b^	39(55.7)	31(44.2)	27(38.5)	43(61.4)	0.042*∗*
During tissue aspiration^b^	55(78.5)	15(21.4)	42(60)	28(40)	0.017*∗*
15 minutes after the procedure^b^	8(11.4)	62(88.5)	7(10)	63(90)	0.785
2 hours after the procedure^a^	3(4.3)	67(95.7)	1(1.4)	69(98.5)	0.31

*∗*Statistic significant: p-value <0.05, VAS: visual analog scale, a: Fisher's exact test, and b: Pearson chi-square.

## Data Availability

The raw data used to support the findings of this study were supplied by Dr.Wiphawee Luangtangvarodom under license and so cannot be made freely available. They are restricted by the ethics board of Faculty of medicine, Thammasat University, Pathumthani,Thailand in order to protect the patients' privacy. Requests for access to these data should be made to Dr. Pongrojpaw for researchers who meet the criteria for access to confidential data.
